# Mitochondrial genomes of the early land plant lineage liverworts (Marchantiophyta): conserved genome structure, and ongoing low frequency recombination

**DOI:** 10.1186/s12864-019-6365-y

**Published:** 2019-12-09

**Authors:** Shanshan Dong, Chaoxian Zhao, Shouzhou Zhang, Li Zhang, Hong Wu, Huan Liu, Ruiliang Zhu, Yu Jia, Bernard Goffinet, Yang Liu

**Affiliations:** 1 Laboratory of Southern Subtropical Plant Diversity, Fairy Lake Botanical Garden, Shenzhen & Chinese Academy of Sciences, Shenzhen, 518004 China; 2 State Key Laboratory for Conservation and Utilization of Subtropical Agro-Bioresources, South China Agricultural University, Guangzhou, 510642 China; 3 Department of Biology, School of Life Sciences, East China Normal University, Shanghai, 200241 China; 4 BGI-Shenzhen, Shenzhen, 518083 China; 5 State Key Laboratory of Systematic and Evolutionary Botany, Chinese Academy of Sciences, Institute of Botany, Beijing, 100093 China; 6 Department of Ecology and Evolutionary Biology, University of Connecticut, Storrs, CT 06269-3043 USA

**Keywords:** Bryophytes, recombination, structural evolution, repeats, introns

## Abstract

**Background:**

In contrast to the highly labile mitochondrial (mt) genomes of vascular plants, the architecture and composition of mt genomes within the main lineages of bryophytes appear stable and invariant. The available mt genomes of 18 liverwort accessions representing nine genera and five orders are syntenous except for *Gymnomitrion concinnatum* whose genome is characterized by two rearrangements. Here, we expanded the number of assembled liverwort mt genomes to 47, broadening the sampling to 31 genera and 10 orders spanning much of the phylogenetic breadth of liverworts to further test whether the evolution of the liverwort mitogenome is overall static.

**Results:**

Liverwort mt genomes range in size from 147 Kb in Jungermanniales (clade B) to 185 Kb in Marchantiopsida, mainly due to the size variation of intergenic spacers and number of introns. All newly assembled liverwort mt genomes hold a conserved set of genes, but vary considerably in their intron content. The loss of introns in liverwort mt genomes might be explained by localized retroprocessing events. Liverwort mt genomes are strictly syntenous in genome structure with no structural variant detected in our newly assembled mt genomes. However, by screening the paired-end reads, we do find rare cases of recombination, which means multiple concurrent genome structures may exist in the vegetative tissues of liverworts. Our phylogenetic analyses of the nuclear encoded double stand break repair protein families revealed liverwort-specific subfamilies expansions.

**Conclusions:**

The low repeat recombination level, selection, along with the intensified nuclear surveillance, might together shape the structural evolution of liverwort mt genomes.

## Background

Mitochondrial (mt) genomes of vascular plants are highly variable in size (i.e., from ca. 66 Kb in *Viscum scurruloideum* [1] to 11.3 Mb in *Silene conica* [2]), and in gene content (i.e., 13 to 64 [3], excluding duplicated genes and open reading frames (ORFs)). By contrast, the mitogenomes within the three bryophyte lineages are conserved in terms of gene content, and exhibit rather narrow size variation [4]. The 40 mt genomes from 29 moss genera (e.g., [5, 6]) are typically smaller (101–141 Kb, median ~ 107 Kb) than those of other land plants, and constantly encode 40 protein-coding genes (PCGs), 24 tRNAs, and 3 rRNAs. The mt genomes of hornworts sampled from four genera [7–9] are larger in size (185–242 Kb) but smaller in gene content (21–23 PCGs, 18–23 tRNAs, 3 rRNAs). The 18 mt genomes from seven liverwort genera [10–14] are intermediate in size between those of mosses and hornworts (142–187 Kb, median ~ 164 Kb) and may comprise more genes (i.e., 39–42 PCGs, 25–27 tRNAs, and 3 rRNAs).

Vascular plant mt genomes contain a variable set of introns, ranging in numbers from three (*Viscum album* [3]) to 37 (*Selaginella moellendorffii* [15]), with an average of 21. Bryophyte mt genomes tend to hold more introns than those of vascular plants, i.e., 28–38 (average 33) in hornworts [7], 26 or 27 in mosses [5], and 23–30 (average 28) in liverworts [13]. Each major land plant lineage contains a number of unique mt introns, and not a single intron is shared across all mt genomes of land plants [16]. As a result, the intron content is conserved within but not among land plant lineages [13, 16], suggesting that vast intron gains and losses happened during the early evolution of land plants, and conservative evolution maintained their stable content in the descendant lineages. Although each bryophyte group appears to hold a stable set of introns that parallels their conserved evolution of mt genomes of overall structure, recent small-scale mitogenomic studies provided evidences for distinct intron losses in leafy liverworts, and suggested retroprocessing as the likely causes [13]. Comprehensive studies with expanded taxon samplings are still needed to provide the framework for reconstructing the evolution of introns in liverwort mt genomes, and to assess the underlying causes for their variation in number.

Mt genomes of vascular plants hold abundant repeated sequences, including some large repeats (> 1000 bp), more medium sized repeats (100–1000 bp), and numerous small repeats (50–100 bp). Increased repeat length and identity facilitate intragenomic recombinations [16], and may hence account for the fluid genome structure of vascular mt genomes. In contrast, the mt genomes of the three bryophyte lineages usually have fewer repeated sequences and are structurally more stable. The mt genome of mosses contains only few and moreover small repeated sequences and is hence structurally nearly static [5]. Hornworts contain relatively more repeated sequences, which may explain the few rearrangements distinguishing the four assembled mitogenomes [7]. Finally, the mt genome of liverworts contains repeated sequences of intermediate abundance and size between those of mosses and hornworts [10]. The structure of their mt genome is highly conserved except for that of *Gymnomitrion concinnatum* [11], for which two inversions are needed to restore collinearity with the other liverwort mt genomes.

The nuclear encoded double-strand break repair (DSBR) proteins are involved in the suppression of the recombination between repeated DNA sequences [17]. Mutation of DSBR proteins in model organisms, such as *Arabidopsis* and *Physcomitrella*, often results in increased rearrangements between repeated sequences in organellar genomes that can impact the function of plastids and/or mitochondria [18–21]. The evolution of plant mt genome structure is therefore shaped by intrinsic parameters, such as repeat-mediated recombination, and extrinsic ones, such as nuclear DSBR proteins [17]. The structure of the mt genome of bryophytes is considered highly conserved and stable [5] but the possible underlying causes such as the repeat recombination level and the evolution of the nuclear encoded DSBR genes remain unexplored. At present, only representatives of five of the currently recognized 15 orders of liverworts have seen their mitogenome assembled [22]. Here we broadened the sampling to exemplars of 10 orders to 1) test whether the mt gene content and 2) the structure of the mt genome is conserved across a broader phylogenetic breadth of liverworts; 3) investigate the repeat recombination rates in liverwort mitochondrion; 4) explore the possible intrinsic and extrinsic factors responsible for the structural evolutionary pattern of liverwort mt genomes.

## Results and Discussion

### Liverwort mitogenomes are small in size but abundant in genes

Assemblies of high throughput sequences from total DNA extracts yielded complete circular mt genomes for 29 liverworts, representing 27 genera, 23 families, and 10 orders, of which five orders were newly sampled for their mt genomes. The sequencing depth ranges from 98 to 756 × (Additional file 1: Table S1). Including the published mt genomes from 13 species, complete liverwort mt genomes are thus available from 37 species, 31 genera, and 10 orders (Additional file 2: Table S2).

**Fig. 1:**
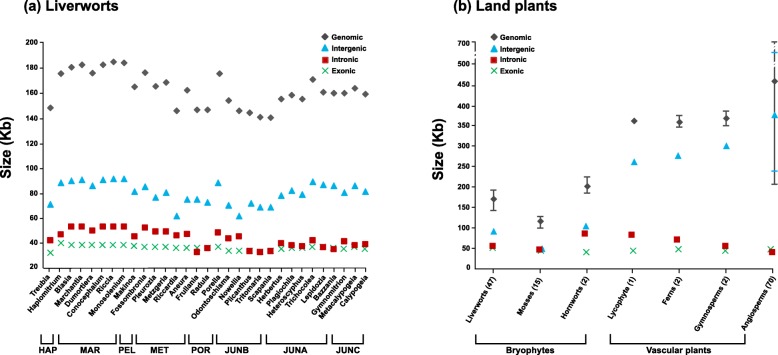
Comparison of mt genome size, intergenic, intronic, and exonic contents among (**a**) liverwort genera, and (**b**) land plant major lineages. The order of liverworts and land plants in (**a**) and (**b**) are sorted by the phylogeny in Fig. 3. Major liverwort clades, HAP (Haplomitriopsida), MAR (Marchantiopsida), PEL (Pelliidae), MET (Metzgeriidae), POR (Porellales), JUNA (Jungermanniales clade A), JUNB (Jungermanniales clade B), JUNC (Jungermanniales clade C), are indicated in (**a**). The number of taxa analyzed for each group is indicated in the bracket following each group’s name in (**b**)

On average, liverwort mt genomes are smaller (~ 167 Kb) than those of vascular plants (average, ~ 476 Kb) or hornworts (average, ~ 197 Kb), but larger than those of mosses (average, ~ 107 Kb). Among liverworts, complex thalloids hold the largest (average, ~ 185 Kb) mt genomes, about 1.3 times the size of those of the Jungermanniopsida clade B that have the smallest mt genomes (average, ~ 147 Kb). Thus, variation in liverwort mitogenome size is much narrower compared to that in angiosperms (i.e., ~ 200-fold range). The trend in the evolution of mitogenome size during the diversification of liverworts is ambiguous (Fig. 1a), compared to a distinct increase during the evolution of land plants (Fig. 1b). The rather stable exome is consistently the smallest component (~ 23%) of liverwort mt genomes (~ 37 Kb). Consequently, changes in total mitogenome size in liverworts are shaped by the variation in intron content and, as shown in other land plants, intergenic spacer size (Fig. 1b).

The mitochondrial genome of liverworts spanning a broad phylogenetic breadth (Additional file 3: Figure S1) holds a nearly constant and large set of PCGs (39–43, Additional file 4: Figure S2). *Treubia* possesses the smallest set of mt PCGs (i.e., 39) among liverworts, and lacks all Cytochrome c genes (*ccmB*, *ccmC*, *ccmFN* and *ccmFC*), a characteristic first reported by Liu et al. [23] and confirmed here based on a new accession. A complete set of Cytochrome c genes is also lacking in the mitogenome of hornworts [7], lycophytes [24], and algae [16]. The *nad7* gene is widespread among algae, mosses, and vascular plants, but is pseudogenized in liverworts, except in the Haplomitriopsida (*Haplomitrium* and *Treubia*) that hold the intact and potentially functional *nad7*, lending support to the hypothesis of the earliest divergence of this lineage in the diversification of liverworts [25]. Ribosomal protein genes have been transferred from the mitochondria to the nucleus many times during the evolution of angiosperms [26]. By contrast, all liverwort mt genome share an identical set of ribosomal protein genes, including *rps8* and *rps10* that rarely occur in other land plant mt genomes (Additional file 5: Table S3). Liverwort mt genomes also encode a rich set of RNA genes among land plants (Additional file 6: Figure S3), including three rRNA genes (*rrn5*, *rrn18* and *rrn26*) and 25–27 tRNA genes (i.e., ± *trnRucg* and *trnTggu*). Among land plants (Additional file 5: Table S3), *trnRucg* is restricted to the complex thalloid liverworts, and *trnTggu* occurs in only few liverwort lineages (i.e., *Blasia*, *Marchantia* and *Haplomitrium*), as well as in mosses and hornworts. Overall, the gene content of mitogenomes seems to be highly conserved during the evolution of liverworts, which spans for at least 400 million years [27].

### Intron content of liverwort mitogenomes is much variable than that of mosses

**Fig. 2:**
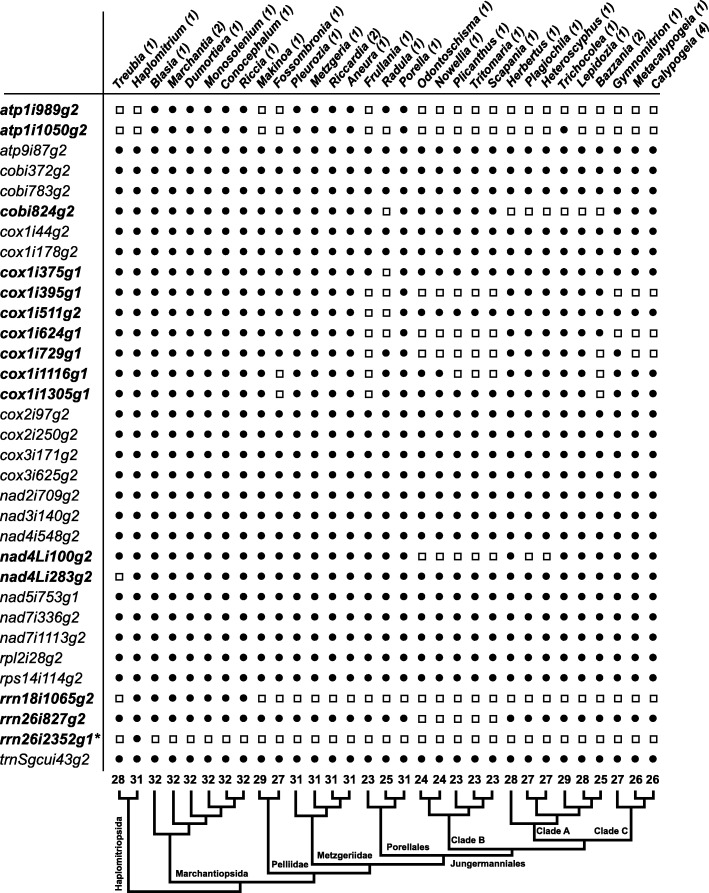
Intron content of liverwort mitochondrial genomes. The species are ordered as in the phylogeny in Fig. 3. The black circle indicates the presence of an intron; the white square the absence of an intron. Intron nomenclature follows Dombrovska and Qiu (2004) and Knoop (2004). The number of species analyzed for each genus is indicated in the bracket following each genus’s name. The numbers at the bottom of each column indicate the total intron number

Liverwort mt genomes hold a rich intron set, ranging from 23 introns in some leafy species to 32 in all the complex thalloid species, with an average of 28 introns (Fig. 2). Species of Haplomitriopsida and Jungermanniopsida possess an average of 29 and 25 introns, respectively. Simple thalloid Pelliidae contains an average of 28 introns, whereas Metzgeriidae hold an average of 31 introns. Leafy Jungermanniidae show most cases of intron losses, especially in the mt genomes of *Frullania*, *Radula*, clade B and C of Jungermanniales (Fig. 2). Among the 17 genes disrupted by at least one intron, six (i.e., *atp1*, *cob*, *cox1*, *nad4L*, *rrn18*, and *rrn26*) vary in their intron content (Fig. 2). The three introns of *cob* gene are commonly present in liverworts, except for 3′ end intron *cobi824*g2 that is absent from *Radula* and clade A of Jungermanniales. The nine introns of *cox1* gene are shared by all thalloid liverworts, and the first three at the 5′ end (*cox1i44*g2, *cox1i178*g2, and c*ox1i375*g1) by almost all liverworts, but the remaining six variably lost among leafy liverworts. The respiratory chain complex I (or *nad*) genes possess a stable intron content, except for *nad4L*, for which one intron (*nad4Li100g2*) is lacking in a few Jungermanniales (clade B, and two species from clade A: *Plagiochila*, *Heteroscyphus*), and another one (*nad4Li283g2*) in *Treubia*. The liverwort unique *rrn18* group II intron (*rrn18i1065*g2) is present only in *Haplomitrium* and complex thalloids. Two introns were found in the ribosomal gene *rrn26*, and intron *rrn26i827*g2 is present in all liverworts but clade B of the Jungermanniales, and *rrn26i2352*g1, a group I intron, is here newly reported and only observed in *Haplomitrium*.

Based on our phylogeny reconstructed from a concatenated nucleotide sequences of 41 mitochondrial protein-coding genes (Fig. 2), the early diverging lineages Haplomitriopsida and Marchantiopsida tend to hold a more complete set of introns compared to the derived clades (especially clade B of Jungermanniales) that seem to have undergone parallel reduction in intron content. The alignments (Additional file 7: Figure S4; Additional file 8: Figure S5) of the four protein coding genes with intron number variations and RNA editing site distributions in liverworts support localized retroprocessing events [13] as the most possible causes for the intron loss phenomenon observed in liverworts. First, all exons are intact and the lost introns appear to be precisely cut off from the splicing site in liverwort mt PCGs. Moreover, liverwort intron losses are more frequent toward the 3′ end of genes (i.e., *cob*, *cox1*), which is a strong characteristic of retroprocessing induced intron losses [28]. Furthermore, in some cases, the intron losses in liverworts seem to be accompanied by RNA editing site losses near the exon-intron splicing boundaries.

### Intergenic spacer variations mirror the genome size variations in liverwort mitogenomes

Intergenic spacers compose the bulk of the mt genome of land plants, accounting, for example, for about 80% in vascular plant mt genome size [16]. Intergenic spacers comprise repeated sequences [29], sequences transferred from plastid [30] or nuclear genomes [31], and DNA fragments horizontally transferred from foreign donors [32, 33]. In liverworts, the intergenic spacers constitute the largest portion of mt genomes (average, ~ 81 Kb, ~ 49%), matching the combined exonic (~ 37 Kb, ~ 24%) and intronic (~ 44 Kb, ~ 27%) components (Table 1). The pattern in spacer length variation parallels that of genome size variation in liverworts (Fig. 1a). On average the complex thalloid mt genomes hold the largest spacer component (~ 91 Kb or ~ 49%), followed by that of *Haplomitrium* (~ 89 Kb or ~ 50%). The average spacer size decreases to ~ 83 Kb (~ 48%) in Pelliidae, 79 Kb (~ 50%) in Porellales, and ~ 74 Kb (~ 45%) in Metzgeriidae. In the Jungermanniales, the average spacer size is relatively small but varies between 84 Kb (~ 51%), 69 Kb (~ 46%), and 82 Kb (~ 51%) in clade A, B, C, respectively (Additional file 2: Table S2).

**Table 1:** Statistics of 47 available liverwort mitochondrial genomes. Taxa are sorted alphabetically. All ratios are calculated to the percentage of spacer length unless otherwise indicated. ORF = open reading frame; SSR = simple sequence repeat

Taxon	GenBank No.	Total spacer size (bp) (% of whole genome)	ORF (%)	Repeats (%)^a^	SSR (%)	Plastid origin (%)	Nuclear origin (%)^b^	Pseudogene (%)
*Aneura pinguis*	NC_026901	75,732 (45.73)	20.87	2.70	0.0	0	15.00	6.4
*Aneura pinguis*	KR817582	75,139 (45.54)	19.60	2.91	0.1	0	15.10	5.7
*Aneura pinguis*	KU140427	74,612 (45.48)	21.70	2.33	0.1	0	15.00	6.2
*Aneura pinguis*	KY242386	74,865 (45.46)	20.38	2.45	0.1	0	16.10	5.9
*Aneura pinguis*	KY702722	76,316 (46.01)	20.30	2.38	0.2	0	14.60	6.1
*Aneura pinguis*	KY702723	77,158 (46.19)	19.62	2.73	0.0	0	15.30	6.3
*Aneura pinguis*	MK230925	77,098 (46.19)	20.79	2.73	0.0	0	15.37	6.3
*Bazzania japonica*	MK230926	86,267 (53.05)	16.42	3.82	0.4	0	17.72	6.2
*Bazzania tridens*	MK318534	86,304 (53.06)	16.07	3.93	0.4	0	17.70	6.2
*Blasia pusilla*	MK230927	90,173 (48.76)	10.94	6.38	0.0	0	28.30	7.3
*Calypogeia arguta*	NC_035978	79,802 (50.17)	8.56	1.94	0.1	0	17.10	5.7
*Calypogeia integristipula*	NC_035977	82,607 (50.66)	13.16	3.56	0.1	0	19.00	6.7
*Calypogeia neogaea*	NC_035980	82,896 (51.12)	14.71	3.47	0.1	0	19.30	6.5
*Calypogeia suecica*	NC_035979	82,723 (51.08)	15.28	3.53	0.2	0	17.70	6.6
*Conocephalum conicum*	MK230928	91,366 (49.08)	12.35	6.09	0.1	0	28.93	6.3
*Dumortiera hirsuta*	MK230929	86,433 (48.56)	15.28	5.36	0.2	0	28.21	6.9
*Fossombronia cristula*	MK230936	85,625 (47.66)	14.22	4.25	0.1	0	20.98	6.6
*Frullania orientalis*	MK230937	75,479 (50.53)	12.11	4.34	0.3	0	19.59	4.4
*Gymnomitrion concinnatum*	NC_040132	80,799 (49.70)	11.92	3.93	0.1	0	18.20	5.3
*Haplomitrium mnioides*	MK230931	89,333 (49.96)	26.24	2.71	0.0	0	12.81	5.3
*Haplomitrium mnioides*	MK230932	89,333 (49.96)	26.24	2.71	0.0	0	12.81	5.3
*Herbertus ramosus*	MK230946	78,882 (49.82)	12.79	4.19	0.2	0	22.08	8.5
*Heteroscyphus zollingeri*	MK230947	79,048 (50.09)	12.92	2.52	0.3	0	16.37	6.4
*Lepidozia trichodes*	MK230954	87,006 (53.25)	20.83	3.53	0.2	0	15.78	6.7
*Makinoa crispata*	MK230958	81,459 (48.73)	19.35	6.61	0.1	0	24.83	7.3
*Marchantia paleacea*	NC_001660	91,942 (49.27)	14.04	7.61	0.1	0	30.90	7.0
*Marchantia polymorpha subsp. ruderalis*	NC_037508	91,328 (49.05)	12.53	7.61	0.1	0	30.79	7.1
*Metacalypogeia alternifolia*	MK230953	86,713 (51.99)	18.18	3.36	0.2	0	17.70	7.3
*Metzgeria leptoneura*	MK230956	80,637 (47.11)	18.08	3.70	0.1	0	18.26	6.6
*Monosolenium tenerum*	MK230931	92,474 (49.29)	15.15	6.82	0.1	0	29.42	6.8
*Monosolenium tenerum*	MK230932	92,476 (49.29)	15.15	6.80	0.1	0	29.42	6.8
*Nowellia curvifolia*	MK230952	61,971 (41.83)	11.66	2.93	0.1	0	14.34	6.7
*Odontoschisma grosseverrucosum*	MK230951	70,494 (45.28)	17.43	3.08	0.2	0	16.56	6.6
*Plagiochila subtropica*	MK230948	82,736 (51.26)	14.44	3.21	0.3	0	17.17	6.5
*Pleurozia purpurea*	MK230956	77,352 (45.90)	16.28	4.20	0.1	0	20.48	7.3
*Pleurozia purpurea*	NC_013444	77,347 (45.90)	15.81	4.19	0.1	0	20.50	7.3
*Plicanthus hirtellus*	MK230959	72,309 (49.32)	16.99	4.38	0.2	0	19.87	6.8
*Porella plumosa*	MK230938	88,966 (49.80)	16.81	5.63	0.1	0	21.08	6.6
*Radula japonica*	MK230939	73,160 (48.90)	20.68	4.42	0.3	0	15.13	5.7
*Riccardia latifrons*	MK230955	61,901 (41.62)	16.47	2.30	0.2	0	15.86	6.0
*Riccardia planiflora*	MK318535	62,537 (41.87)	18.13	1.89	0.11	0	14.90	5.1
*Riccia cavernosa*	MK230934	92,483 (49.23)	12.37	6.77	0.1	0	29.45	6.4
*Scapania ornithopodioides*	MK230950	68,944 (48.22)	11.81	3.40	0.1	0	17.42	7.3
*Treubia lacunosa*	NC_016122	71,724 (47.45)	16.60	2.32	0.0	0	18.55	6.1
*Treubia lacunosa*	MK230943	72,567 (47.75)	15.50	2.26	0.0	0	18.60	6.1
*Trichocolea tomentella*	MK230949	89,913 (51.74)	16.73	3.62	0.8	0	18.75	6.3
*Tritomaria quinquedentata*	MG640570	69,393 (48.69)	15.19	3.10	0.2	0	21.10	6.2

^a^Repeated sequences were detected by BLASTN, and sequences with similarity higher than 85% and aligned length longer than 50 bp were considered as repeat

^b^Putative nuclear-derived sequences were detected by blasting the whole-intergenic spacer of each species against the *Marchantia polymorpha* nuclear genome; blast hits with E value 0.00001 were summarized. For length calculation, overlapping regions in mitochondrial spacers were removed

The spacer region of liverwort mt genomes is composed of ORFs, pseudogene fragments, nuclear homologous sequences, dispersed repeated sequences, SSRs, and other non-coding sequences of unknown origin. Plastid-derived or horizontally transferred DNA sequences are seemingly lacking in liverwort mt genomes (Table 1), as they were in those of mosses [5]. Nuclear derived sequences generally make up the largest component with an average of 20%, ranging from 13% in *Haplomitrium* to over 30% in the Marchantiopsida. Considering that we used the nuclear genome of *Marchantia polymorpha*, the only available nuclear genome for liverworts, as the reference for the blast search, it is not surprising that *Marchantia polymorpha* mt spacers returned the highest percentage hit (31%) on homologous sequences. The fast evolving/long branch lineage *Haplomitrium* (13%) and some simple thalloid taxa (such as *Aneura* and *Riccardia*) show the lowest percentage (i.e., 15%) of homologous sequence hits. Generally, the total size of the nuclear homologous sequence content in liverworts (average, ~ 15.7 Kb) is comparable to that in mosses (average, ~ 14.7 Kb), but the percentage relative to the total spacer size is only half that of mosses (average, ~ 42% [5]), which reflects the smaller size of moss mt spacers. ORFs usually make up the second largest part of the liverwort mt spacers, with an average ratio of 16%, ranging from 12% in some Jungermanniales species to 26% in *Haplomitrium*. The ratio of ORF to spacer size is smallest in the Marchantiopsida (average, ~ 13%), followed by the Jungermanniopsida (average, ~ 15%), then simple thalloids (average, ~ 17%). The ORF content in liverworts is distinctively larger than in mosses (i.e., 5%). As in mosses, SSR sequences compose generally less than 1% of the combined spacer region in liverwort mt genomes. Whereas moss mitogenomes lack repeated sequences in their intergenic spacers, liverworts hold on average 4% of repeated sequences in their intergenic spacer regions, with only ~ 2% in the Haplomitriopsida and ~ 7% in the Marchantiopsida. The relatively higher repeated sequence content might allow for higher potential for mt genome recombination, as a positive correlation between the number of repeated sequences and the gene rearrangements has been suggested [5].

### Liverwort mitogenomes are conserved in gene order despite repeat mediated recombinations

**Fig. 3:**
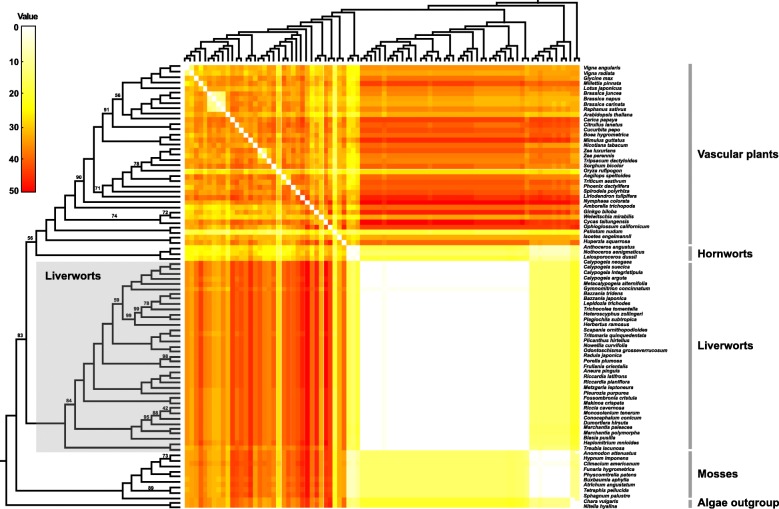
Heat map of mt genome rearrangements in pairwise comparisons of land plants along a phylogenetic tree based on mt nucleotide sequences (Additional file 3: Figure S1). All nodes are maximally supported (i.e., 100% bootstrap support) unless otherwise marked

Repeated sequences play an important role in plant mt genome structure stability since they can mediate intragenomic homologous recombinations leading to inversions and translocations of genomic regions [1, 16, 34]. Except for the mitogenome of *Gymnomitrion concinnatum* of the Jungermanniales [11], all remaining available liverwort mt genomes (i.e., 46) share exactly the same gene order (Fig. 3). The mt genome of *G. concinnatum* needs two inversions to make it collinear with the other liverwort mt genomes, which is in stark contrast to the situation in vascular plants wherein any two mt genomes require on average 31 rearrangements to gain collinearity [5]. The stable mt genome structure of liverworts might suggest either very low recombination level in liverwort mitochondrion and/or intensified nuclear surveillance over repeat recombinations.

Compared to the compact moss mt genomes with only a few small repeats shorter than 100 bp, liverwort mt genomes are much inflated, containing on average 14 pairs of small repeated sequences of 50–100 bp, and 14 pairs of medium-sized repeats of 100–900 bp (Additional file 9: Table S4). We examined the recombination rates of all repeats (535 pairs, Additional file 10: Table S5) within the 50–250 bp range for all the 29 samples and found recombination evidence for 26 repeats from 16 species (Table 2), 40% of the liverwort species show no evidence of recombination. The mitogenomes of nine leafy liverworts appear to recombine more frequently than those of complex thalloids (three species) and simple thalloids (two species). The two representatives of the early diverging Haplomitriopsida have recombinants detected. The repeats actively involved in recombination have an average size of the 103 bp with ten repeats (38%) exceeding 100 bp in length. Recombination rates range from 0.67 to 60% with a median value of 4.45%. About two thirds of the recombination events were mediated by small repeats (i.e., shorter than 100 bp) with a median recombination rate of 11.46%. Repeat length and recombination rate are not positively correlated. About half (12 out of 26) of the repeat mediated recombinations cause gene order changes and direct repeat recombinations affect genes more often than their inverted counterparts (62.5% vs 30%). Most (eight out of 10) inverted and four out of 16 direct repeat recombinations cause gene order changes. These gene order changes could give rise to alternative genome conformations, which, if they occurred in germ cells would be passed on to the offspring [35].

**Table 2:** Recombination frequency of repeats within 50–250 bp range for 16 liverwort mitogenomes

Species	Length (bp)	Identity (%)	Direction	Position	Reads support master circle conformation	Reads support alternative conformation	Gene order state after recombination	Genes affected
*Bazzania japonica*	51	94.12	direct	142,103–142,153	30 (68.18%)	14 (31.82%)	unchanged	*trnSUGA*
				141,693–141,743				
	66	87.88	direct	70,639–70,704	39 (84.78%)	7 (15.22%)	unchanged	*atp8*
				70,053–70,118				
*Conocephalum conicum*	138	93.48	direct	85,167–85,304	148 (99.33%)	1 (0.67%)	unchanged	*cox2* and *cox3*
				84,355–84,492				
*Fossombronia cristula*	73	98.63	inverted	60,943–61,014	67 (57.76%)	49 (42.24%)	unchanged	–
				58,015–58,087				
	95	86.32	direct	45,965–46,059	67 (60.91%)	43 (39.09%)	unchanged	–
				46,130–46,221				
	93	87.1	inverted	61,247–61,339	86 (81.9%)	19 (18.1%)	unchanged	–
				58,038–58,130				
	52	96.15	direct	55,570–55,619	80 (97.57%)	2 (2.43%)	unchanged	–
				55,443–55,494				
	73	98.63	direct	61,341–61,413	80 (98.73%)	1 (1.23%)	unchanged	–
				60,943–61,014				
*Frullania orientalis*	75	96	inverted	12,671–12,745	12 (92.31%)	1 (7.69%)	changed	–
				12,671–12,745				
*Haplomitrium mnioides* I	77	88.31	direct	87,784–87,860	48 (97.96%)	1 (2.04%)	changed	*cox3* and *trnSGCU*
				40,562–40,637				
	241	100	direct	140,823–141,063	95 (98.96%)	1 (1.04%)	changed	–
				132,233–132,473				
	51	90.2	direct	14,195–14,245	35 (97.2%)	1 (2.8%)	unchanged	–
				14,085–14,135				
*Heteroscyphus zollingeri*	253	96.05	direct	66,972–67,223	27 (96.43%)	1 (3.57%)	changed	*rps7*
				21,339–21,591				
*Monosolenium tenerum* I	138	92.03	direct	86,651–86,787	94 (98.95%)	1 (1.05%)	unchanged	*cox2* and *cox3*
				85,840–85,977				
*Nowellia curvifolia*	120	100	inverted	88,658–88,777	31 (60.78%)	20 (39.22%)	changed	*cob*
				5529–5648				
	138	98.55	inverted	88,962–89,099	62 (78.48%)	17 (21.52%)	changed	*cob*
				5392–5529				
	64	93.75	inverted	14,0191–14,0253	30 (96.77%)	1 (3.23%)	changed	–
				14,0191–14,0253				
*Odontoschisma grosseverrucosum*	52	94.23	inverted	94,954–95,005	32 (40%)	48 (60%)	changed	*cob*
				5637–5688				
*Pleurozia purpurea*	140	98.57	direct	113,528–113,667	21 (95.46%)	1 (4.54%)	unchanged	*atp1*
				108,644–108,788				
*Plicanthus hirtellus*	54	98.15	inverted	112,052–112,105	31 (48.44%)	33 (51.56%)	changed	–
				84,436–84,489				
*Porella plumosa*	62	90.32	inverted	45,426–45,485	20 (95.24%)	1 (4.76%)	changed	–
				45,426–45,485				
*Radula japonica*	114	89.47	direct	68,120–68,227	49 (98%)	1 (2%)	unchanged	*cox2* and *cox3*
				67,321–67,434				
	189	99.47	direct	142,227–142,415	67 (98.53%)	1 (1.47%)	changed	–
				79,402–79,590				
*Riccia cavernosa*	77	87.01	inverted	110,110–110,185	66 (95.65%)	3 (4.35%)	changed	–
				110,110–110,185				
*Treubia lacunosa*	137	93.43	direct	72,748–72,884	82 (98.8%)	1 (1.2%)	unchanged	*cox2* and *cox3*
				72,001–72,137				
*Trichocolea tomentella*	63	92.06	direct	151,893–15,1955	14 (82.35%)	3 (17.65%)	unchanged	*trnSUGA*
				151,475–15,1537				

Although empirical studies suggest that repeats longer than 50 bp and with an identity above 85% may mediate recombinations [17, 34], the recombination activity of repeats is actually positively correlated with the length of repeated sequences, with small repeats (< 100 bp) rarely inducing recombinations [36, 37]. The detection of repeat recombinations for two thirds of liverwort species with an average of two active repeats per species might indicate repeat recombinations occurred, but in low frequencies. Considering the average of five repeats (per species) longer than 250 bp not investigated for recombinations, it is very likely that some of these larger repeats (250–900 bp) may also allow for recombination. As recombination and structure fluidity are supposed to be positively correlated [1, 4, 5], the stable mt genome structure across liverwort diversity that spans over than 400 myr [27] is surprising given that liverwort mt genomes indeed recombine and alternative genome conformations coexisted. The apparent paradox of structural stasis of the mitogenome during the evolution of liverworts despite evidence of ongoing recombination may be addressed in the following three contexts. First, as some authors reported the adaptive value of repeats and recombinations in plant mitochondrion [37], recombinations happen more frequently when the cells are in stress and/or under some environmental stimuli [38], it is likely that the low recombinations observed in liverworts might primarily happen in the old vegetative cells rather than in young differentiating cells and germ cells, therefore the liverwort progeny inherited the master circle conformation. Second, if repeat recombinations occurred in the reproductive cells, those recombinants with genes affected or genes missing might be selected against. Recent studies in *Drosophila* also suggested a generalizable mechanism for selection against deleterious alternative mt configurations: ATP selection after mitochondrion fragmentation can retain those mtDNA fragments that contain the ‘correct’ mt genome with the complete set of the genes [39]. Nevertheless, those alternative conformations with altered gene order but no genes affected may survive this ATP selection and might possibly lead to offspring with rearranged gene order. Finally, It is also possible that maintaining the organization of genes is essential to the transcription of polycistronic operons in liverworts and mosses [5], hence an identical gene order is selected across all liverwort lineages despite the existence of alternative genome conformations.

### Liverwort mt genomes might be under intensified nuclear surveillance

The structural stability of liverwort mt genomes, in accordance with the remarkable structural conservatism of liverwort plastomes [40], might also be shaped by nuclear encoded DSBR proteins that suppress the error-prone ectopic recombinations across small direct repeats during DSBR in mitochondrion and plastid [41]. We here characterized six frequently reported DSBR gene families in the transcriptome assemblies of 125 land plant representatives (Additional file 11: Table S6). Functional studies have confirmed the mtDNA repair function of the moss orthologs in *Physcomitrella patens* for four of these gene families, *RecA*, *RecG*, *RecX*, and *MSH1*. In our phylogenetic analyses (Additional file 12: Figure S6), four DSBR gene families (*RecA*, *RecG*, *RecX*, and *OSB*) show notable liverwort-specific subfamily expansions, suggesting peculiar evolutionary pattern of DNA repair mechanisms in liverworts. The subcellular localization of these DSBR proteins (as predicted by TargetP) yielded ambiguous results for most of these DSBR proteins, including those expanded ones, suggesting either truncated protein sequences or incomplete query database. Although the subcellular localization (as predicted by TargetP), and the in vivo function of these expanded liverwort gene family members remain elusive, we could not rule out the possibility that these liverwort specific expansions might also contribute to the mtDNA sequence stability of liverworts. Specifically, selective pressure might have driven the functional divergence of DSBR gene families under different evolutionary constraints, and that those diversified DSBR proteins may exhibit a wider range of characteristics and specificities, which possibly help intensify the DNA repair mechanisms in liverworts.

## Conclusions

Based on the assembly of mitochondrial genomes for a widely expanded sample of liverworts spanning over 400 million years of evolution [27], we a) confirm that the mitogenome of liverworts is highly conserved in both gene content and gene order, while having a large and variable set of introns (23–32 introns) subject to a series of localized retroprocessing events, b) reveal ongoing intragenomic recombination mediated by small repeats and c) show liverwort specific DSBR subfamily expansions in four gene families. Although the mt genomes are relatively conserved in each of the three major bryophyte lineages (i.e., liverworts, mosses and hornworts), they differ in the content of repeat sequences and frequency of rearrangements, suggesting that the mechanisms maintaining mitogenome structure differ among these lineages. The conserved evolution of liverwort mt genomes might be explained by low recombination level, selection, and intensified nuclear surveillance.

## Methods

### Materials and DNA extractions

Wild samples of liverwort materials of 29 accessions were collected in field trips from China (Xizang, Sichuan, Yunnan, Fujian, and Guangzhou Provinces), Madagascar, the United States (Connecticut), Vietnam, and New Zealand (Additional file 1: Table S1). No specific permissions were needed on current study of these samples. These samples were studied and identified by Qiang He, and the voucher specimens were deposited in SZG (Herbarium of Shenzhen Fairy lake Botanical Garden, Shenzhen, China). Our sampling spreads across the liverwort phylogeny, representing 10 of the 15 orders of extant liverworts. Each sample was cleaned with distilled water and dried using lab paper. The clean shoots of individual samples were isolated under the dissecting microscope and used for DNA extractions using the modified CTAB methods [42]. The DNA quality and quantity of each sample were examined using 1% Agarose gel electrophoresis and Nanodrop 2000 spectrophotometer.

### Mitochondrial genome sequencing and assembly

For genomic DNA sequencing, 1 μg high quality genomic DNA was sheared using the Covaris M220 (Woburn, MA, USA), DNA fragments of 300–500 bp were used to generate sequencing libraries using the Illumina TruSeq™ DNA PCR-free library preparation kit (Illumina, CA, USA) following the manufacturer’s instructions. The libraries were paired-end (2 × 150 bp) sequenced on an Illumina HiSeq 2000 sequencing platform at the WuXiNextCode (Shanghai, China). Approximately 10 Gb sequencing data were generated for each sample. The raw NGS data were trimmed and filtered for adaptors, low quality reads, undersized inserts, and duplicate reads using Trimmomatic [43]. The filtered reads for each species were de novo assembled using CLC Genomics Workbench v5.5 (CLC Bio, Aarhus, Denmark). All assembled contigs were blasted to the *Marchantia polymorpha* mt genome (GenBank accession: NC_001660) to identify mt contigs. As the genomes of the three cellular compartments are significantly different in copy numbers and hence read coverage in sequencing [44], the read depth for mt contigs is distinctly lower than that of plastid contigs, but significantly higher than that of nuclear contigs [45]. For each species, every resulting mt contigs (usually with one to five per species) was first checked for read depth to ensure they are in a similar range, and then each of these mt contig was elongated at both ends in Geneious v10.0.2 (Biomatters, New Zealand) using Bowtie2 [46] till their ends overlapped with one another by at least 5000 bp. Altogether, 29 circular mt genomes were assembled for liverworts. Finally, the corresponding genomic reads were mapped back to the complete mitochondrial genomes to double check for sequencing depth (Additional file 1: Table S1) of the assembled mt genomes. All the 29 liverwort mt genomes newly assembled in this study received constant depth along the mt genomes.

### Genome annotation and comparative analysis

The mt genomes were annotated following the steps described by Li et al. [9] and Xue et al. [8]. In summary, the protein-coding and rRNA genes were annotated by Blastn searches of the non-redundant database of National Center for Biotechnology Information (NCBI). The exact gene and exon/intron boundaries were further verified in Geneious v10.0.2 (Biomatters, New Zealand) by aligning orthologous genes from the published annotated liverwort mitochondrial genomes. The tRNA genes were annotated using tRNAscan-SE v2.0 [47]. Mitochondrial RNA editing sites were obtained from our previous study on liverwort organellar RNA editing [48]. We summarized and compared the structural evolution of all the formally published (as by June 2019) liverwort mt genomes (Additional file 2: Table S2) from the gff3 annotation files. As accessions from the same species/genus show very similar characters in mt genomes in all aspects, directly averaging the values across all organisms would bias towards better-represented lineages. Therefore, to avoid such biases we calculated the average values under three different strategies, if multiple accessions of a species exist: (1) we average them first and then calculate the average value among species (e.g., genome/exon/intron length, and gene/intron number); (2) similarly for among genera, we averaged the values of species, and (3) for among clades, we averaged the value of each genus.

### Repeats and recombination analysis

Repeat sequences are annotated and analyzed (Additional file 9: Table S4) using the python tool developed by Wynn & Christensen [37]. Considering at least 50 bp matches at both ends of the insert fragment and an average size of insert fragments being 350 bp in our sequencing libraries, we can estimate the recombination rate of all non-overlapping repeats between 50 bp and 250 bp. Altogether, we analyzed 534 repeats for the 29 liverwort mt genomes (Additional file 10: Table S5) following Dong et al. [36]. Specifically, for each repeat pair, we built four or eight reference sequences, each with 200 bp up- and down-stream of the two template sequences (original sequences), and the two (for repeat pair with identity = 100) or six (for repeat pair with identity < 100) recombined sequences (alternative configurations) constructed from the putative recombination products. All the recombined templates were blasted against the corresponding mt genome sequences to identify those on the genome. Then, we searched the reference sequences against the corresponding paired-end reads database, and counted the number of matching read pairs with a blast identity above 98%, and a hit coverage of at least 50 bp in both flanking regions of each repeat sequence. After that, the best matched read pairs for all the recombinants were extracted and mapped to the corresponding mt genomes using Bowtie2 [46] and the resultant bam file was visualized in Geneious v10.0.2 (Biomatters, New Zealand) to confirm the existence of the alternative configurations in reads.

### Gene alignment and phylogenetic reconstruction

For the phylogenetic reconstruction, sequences of each of the 41 mitochondrial genes were aligned using TranslatorX [49], which first translates the nucleotide (nt) sequence into amino acids (aa) using the standard, universal genetic code, and then uses MAFFT [50] to create an amino acid alignment. The alignment is further trimmed for ambiguous portions by GBLOCKS [51] with the least stringent settings. The cleaned aa alignment is then used as a guide to generate the nt sequence alignment. The resultant nt alignments of individual genes were then combined as one dataset in Geneious v10.0.2 (Biomatters, New Zealand). The concatenated nt dataset was analyzed by the maximum likelihood (ML) method as implemented in the RAxML software [52] with codon-partitioned GTRGAMMA model. Branch support for each internode was evaluated with 300 bootstrap approximation (Additional file 3: Figure S1). The mitochondrial phylogeny of liverworts is mostly congruent with that of the plastid phylogeny [53].

### Identification of DSBR proteins and phylogenetic analysis

We used HMMER [54] to conduct profile hidden Markov model (HMM) searches using the Pfam *RecA* (PF00154), *SSB* (PF00436), *Whirly* (PF08536), *RecX* (PF02631), *MutS_V* (PF00488), *DEAD* and *Helicase_C* (PF00270, PF00271) domains as queries to search the annotated proteins from 125 plant species (Additional file 11: Table S6) for characterization of the gene family members of *RecA*, *OSB*, *Why*, *RecX*, *MSH*, and *RecG*, respectively. For gene loci with multiple isoforms predicted, the primary isoform was used if primary isoform annotation is available; otherwise the longest protein was used. We considered sequences with Pfam domain identified by HMMER with an E-value of 1e-6, and the hit alignment length of at least 50% of the domain length to be candidate proteins. Those candidate proteins from the HMMER search were further manually confirmed using the SMART [55] and Pfam [56] databases. The proteins sequences were filtered for redundancy using CD-hit [57] with a similarity threshold of 0.95, aligned using MATTF [50], and trimmed for ambiguous portions by GBLOCKS with the least stringent settings [51]. The final protein alignment were analyzed by the maximum likelihood (ML) method as implemented in the IQ-TREE software [58] with 1000 ultrafast bootstrap replicates. The subcellular localizations of these DSBR proteins were predicted using online TargetP 1.1 server [59].

## Supplementary information

**Additional file 1: Table S1.** Overview of the 29 newly generated liverwort mitochondrial genomes.

**Additional file 2: Table S2.** Comparison of the genome content of the 47 liverwort mitochondrial genomes.

**Additional file 3: Figure S1.** ML phylogram of selected land plants with an emphasis on liverworts based on a concatenated nucleotide data set.

**Additional file 4: Figure S2.** Distribution of mitochondrial protein genes in 31 liverwort genera.

**Additional file 5: Table S3.** Comparison of gene content and gene order from 88 selected land plant mitochondrial genomes.

**Additional file 6: Figure S3.** Distribution of mitochondrial RNA genes in 31 liverwort genera.

**Additional file 7: Figure S4.** RNA editing site variations and intron losses in the *cox1* gene as an exemplar.

**Additional file 8: Figure S5.** RNA editing site distributions on three gene alignments in a phylogenetic context.

**Additional file 9: Table S4.** Distribution of repeat sizes among liverwort mitochondrial genomes.

**Additional file 10: Table S5.** Repeat recombinations for 534 repeats within 50 to 300 bp range in liverwort mitochondria.

**Additional file 11: Table S6.** Taxa list for DSBR protein identification.

**Additional file 12: Figure S6.** Phylogenetic trees of DSBR protein sequences from 125 Viridiplantae taxa inferred by Iqtree.

## Data Availability

Newly generated mitochondrial genomes of 29 liverworts have been deposited in GenBank under the accession numbers MK230925–MK230962. The raw genomic NGS read data have been deposited in the Short Read Archive (SRA) database of NCBI under the study number SRP170808. Other supporting results are included within the article and its additional files.

## Abbreviations

aa: Amino acid
DSBR: Double strand break repair
ML: Maximum likelihood
Mt genome: Mitochondrial genome
mtDNA: Mitochondrial DNA
nt: Nucleotide
ORFs: Open reading frames
PCGs: Protein-coding genes
rRNAs: Ribosomal RNAs
SZG: Herbarium of Shenzhen Fairy lake Botanical Garden, Shenzhen, China
tRNAs: Transfer RNAs
