# Supplementary material for: Mitochondrial genomes of the early land plant lineage liverworts (Marchantiophyta): conserved genome structure, and ongoing low frequency recombination
Source: BMC Genomics. 2019 Dec 9;20:953. doi: 10.1186/s12864-019-6365-y (PMC6902596; doi:10.1186/s12864-019-6365-y)
Supplement: Supplementary file 3 — Additional file 3: Figure S1. ML phylogram of selected land plants with an emphasis on liverworts based on a concatenated nucleotide data set. [file 12864_2019_6365_MOESM3_ESM.pdf]

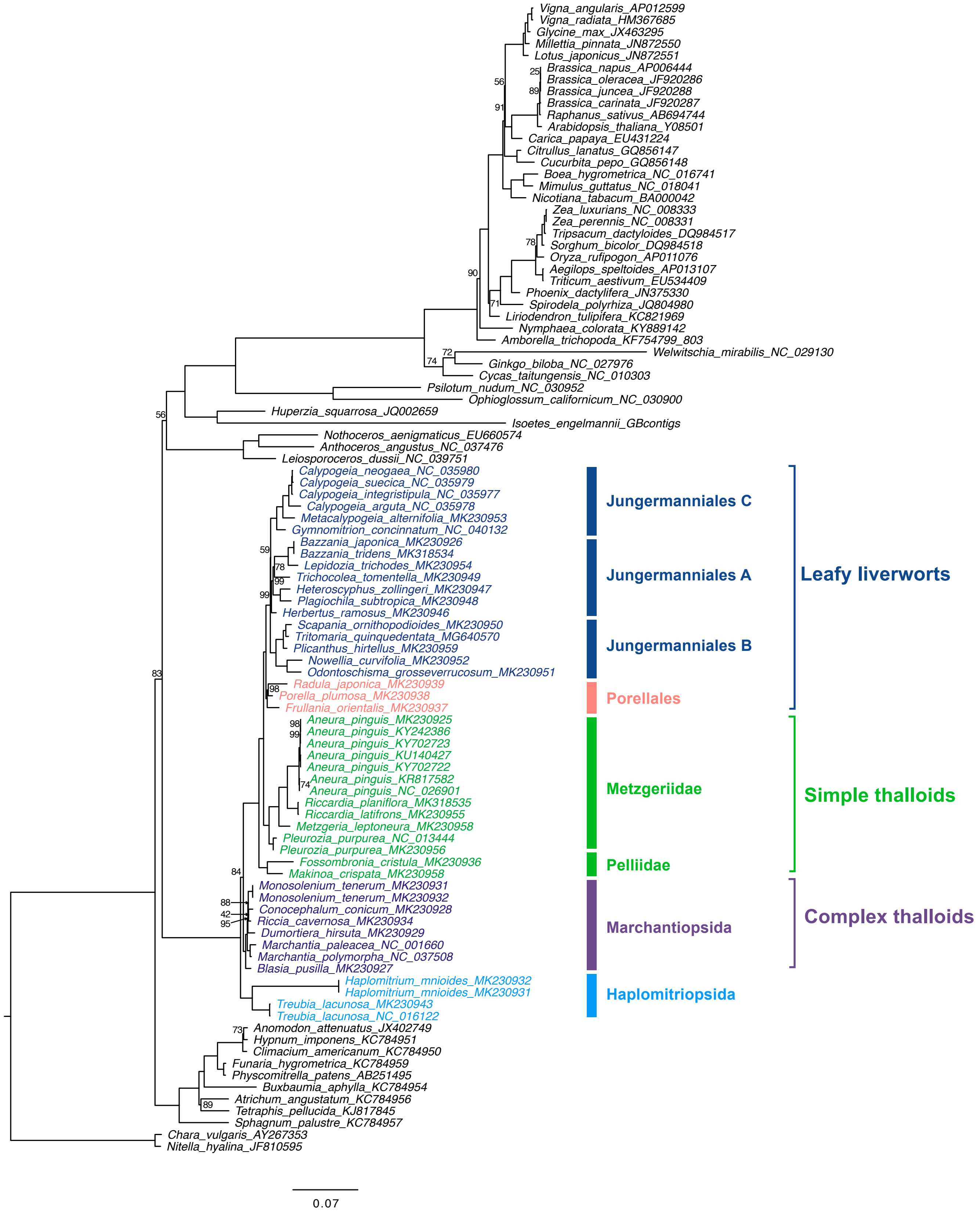

Figure S1. ML phylogram of selected land plants with an emphasis on liverworts based on a concatenated nucleotide data set (all codon positions) of 41 mitochondrial genes, all branches are maximally supported with 300 bts in RAxML unless otherwise marked.
