# Supplementary material for: Mitochondrial genomes of the early land plant lineage liverworts (Marchantiophyta): conserved genome structure, and ongoing low frequency recombination
Source: BMC Genomics. 2019 Dec 9;20:953. doi: 10.1186/s12864-019-6365-y (PMC6902596; doi:10.1186/s12864-019-6365-y)
Supplement: Supplementary file 6 — Additional file 6: Figure S3. Distribution of mitochondrial RNA genes in 31 liverwort genera. [file 12864_2019_6365_MOESM6_ESM.pdf]

	Treubia (1)	Haplomitrium (1)	Blasia (1)	Marchantia (2)	Dumortiera (1)	Monosolenium (1)	Conocephalum (1)	Riccia (1)	Makinoa (1)	Fossombronia (1)	Pleurozia (1)	Metzgeria (1)	Riccardia (2)	Aneura (1)	Frullania (1)	Radula (1)	Porella (1)	Odontoschisma (1)	Nowellia (1)	Plicanthus (1)	Tritomaria (1)	Scapania (1)	Herbertus (1)	Plagiochila (1)	Heteroscyphus (1)	Trichocolea (1)	Lepidozia (1)	Bazzania (2)	Gymnomitrion (1)	Metacalypogeia (1)	Calypogeia (4)
rrn26	●	●	●	●	●	●	●	●	●	●	●	●	●	●	●	●	●	●	●	●	●	●	●	●	●	●	●	●	●	●	●
rrn18	●	●	●	●	●	●	●	●	●	●	●	●	●	●	●	●	●	●	●	●	●	●	●	●	●	●	●	●	●	●	●
rrn5	●	●	●	●	●	●	●	●	●	●	●	●	●	●	●	●	●	●	●	●	●	●	●	●	●	●	●	●	●	●	●
trnAugc	●	●	●	●	●	●	●	●	●	●	●	●	●	●	●	●	●	●	●	●	●	●	●	●	●	●	●	●	●	●	●
trnCgca	●	●	●	●	●	●	●	●	●	●	●	●	●	●	●	●	●	●	●	●	●	●	●	●	●	●	●	●	●	●	●
trnDguc	●	●	●	●	●	●	●	●	●	●	●	●	●	●	●	●	●	●	●	●	●	●	●	●	●	●	●	●	●	●	●
trnEuuc	●	●	●	●	●	●	●	●	●	●	●	●	●	●	●	●	●	●	●	●	●	●	●	●	●	●	●	●	●	●	●
trnFgaa	●	●	●	●	●	●	●	●	●	●	●	●	●	●	●	●	●	●	●	●	●	●	●	●	●	●	●	●	●	●	●
trnGgcc	●	●	●	●	●	●	●	●	●	●	●	●	●	●	●	●	●	●	●	●	●	●	●	●	●	●	●	●	●	●	●
trnGucc	●	●	●	●	●	●	●	●	●	●	●	●	●	●	●	●	●	●	●	●	●	●	●	●	●	●	●	●	●	●	●
trnHgug	●	●	●	●	●	●	●	●	●	●	●	●	●	●	●	●	●	●	●	●	●	●	●	●	●	●	●	●	●	●	●
trnIcau	●	●	●	●	●	●	●	●	●	●	●	●	●	●	●	●	●	●	●	●	●	●	●	●	●	●	●	●	●	●	●
trnKuuu	●	●	●	●	●	●	●	●	●	●	●	●	●	●	●	●	●	●	●	●	●	●	●	●	●	●	●	●	●	●	●
trnLcaa	●	●	●	●	●	●	●	●	●	●	●	●	●	●	●	●	●	●	●	●	●	●	●	●	●	●	●	●	●	●	●
trnLuaa	●	●	●	●	●	●	●	●	●	●	●	●	●	●	●	●	●	●	●	●	●	●	●	●	●	●	●	●	●	●	●
trnLuag	●	●	●	●	●	●	●	●	●	●	●	●	●	●	●	●	●	●	●	●	●	●	●	●	●	●	●	●	●	●	●
trnMcau	●	●	●	●	●	●	●	●	●	●	●	●	●	●	●	●	●	●	●	●	●	●	●	●	●	●	●	●	●	●	●
trnfMcau	●	●	●	●	●	●	●	●	●	●	●	●	●	●	●	●	●	●	●	●	●	●	●	●	●	●	●	●	●	●	●
trnNguu	●	●	●	●	●	●	●	●	●	●	●	●	●	●	●	●	●	●	●	●	●	●	●	●	●	●	●	●	●	●	●
trnPugg	●	●	●	●	●	●	●	●	●	●	●	●	●	●	●	●	●	●	●	●	●	●	●	●	●	●	●	●	●	●	●
trnQuug	●	●	●	●	●	●	●	●	●	●	●	●	●	●	●	●	●	●	●	●	●	●	●	●	●	●	●	●	●	●	●
trnRacg	●	●	●	●	●	●	●	●	●	●	●	●	●	●	●	●	●	●	●	●	●	●	●	●	●	●	●	●	●	●	●
trnRucg	□	□	●	●	●	●	●	●	□	□	□	□	□	□	□	□	□	□	□	□	□	□	□	□	□	□	□	□	□	□	□
trnRucu	●	●	●	●	●	●	●	●	●	●	●	●	●	●	●	●	●	●	●	●	●	●	●	●	●	●	●	●	●	●	●
trnSgcu	●	●	●	●	●	●	●	●	●	●	●	●	●	●	●	●	●	●	●	●	●	●	●	●	●	●	●	●	●	●	●
trnSuga	●	●	●	●	●	●	●	●	●	●	●	●	●	●	●	●	●	●	●	●	●	●	●	●	●	●	●	●	●	●	●
trnTggu	□	●	●	●	□	□	□	□	□	□	□	□	□	□	□	□	□	□	□	□	□	□	□	□	□	□	□	□	□	□	□
trnVuac	●	●	●	●	●	●	●	●	●	●	●	●	●	●	●	●	●	●	●	●	●	●	●	●	●	●	●	●	●	●	●
trnWcca	●	●	●	●	●	●	●	●	●	●	●	●	●	●	●	●	●	●	●	●	●	●	●	●	●	●	●	●	●	●	●
trnYgua	●	●	●	●	●	●	●	●	●	●	●	●	●	●	●	●	●	●	●	●	●	●	●	●	●	●	●	●	●	●	●
	28	29	30	30	29	29	29	29	28	28	28	28	28	28	28	28	28	28	28	28	28	28	28	28	28	28	28	28	28	28	28

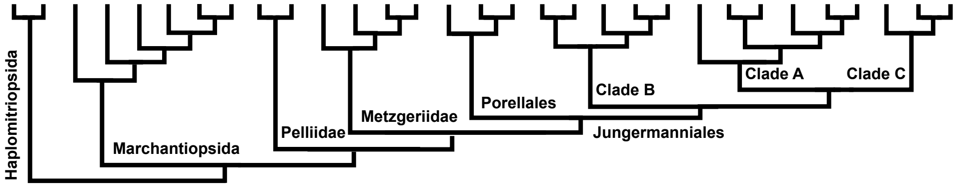

Figure S3. Distribution of mitochondrial RNA genes in 31 liverwort genera. Present (*dot*), absent (*square*). Taxa are sorted according to the topology in Supplementary Figure S1.
