# Supplementary material for: Mitochondrial genomes of the early land plant lineage liverworts (Marchantiophyta): conserved genome structure, and ongoing low frequency recombination
Source: BMC Genomics. 2019 Dec 9;20:953. doi: 10.1186/s12864-019-6365-y (PMC6902596; doi:10.1186/s12864-019-6365-y)
Supplement: Supplementary file 7 — Additional file 7: Figure S4. RNA editing site variations and intron losses in the cox1 gene as an exemplar. [file 12864_2019_6365_MOESM7_ESM.pdf]

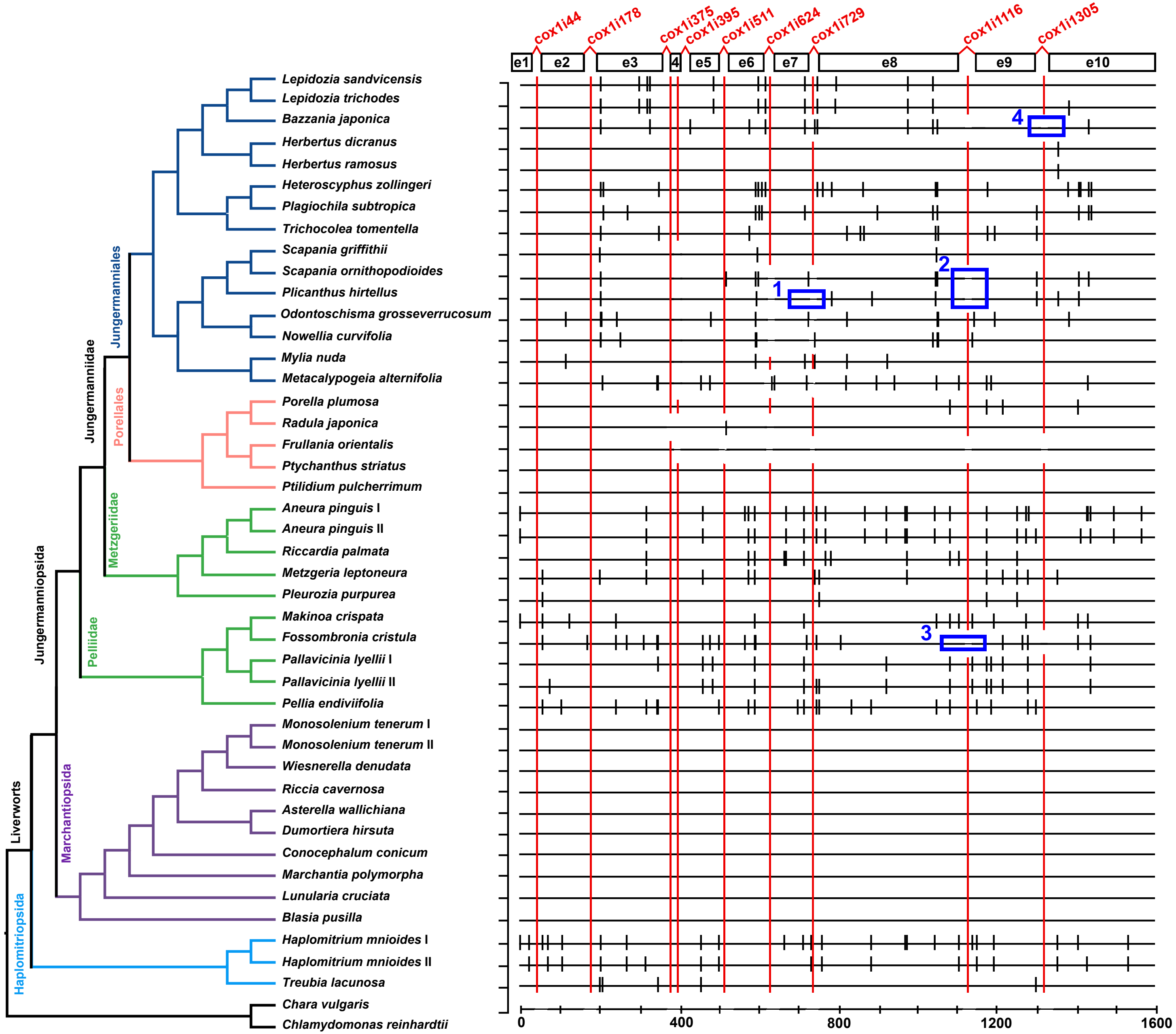

Figure S4. RNA editing site variations and intron losses in the cox1 gene as an exemplar. The red lines indicate the positions of intron insertions, and the short black lines indicate the positions of empirical RNA editing sites. The blue boxes indicates the evidences of retroprocessing. The phylogeny used here is based on 128 organellar genes (Dong et al. Unpublished). These RNA editing data can be obtained from the draft mitochondrial genomes of liverworts deposited in the China National GeneBank DataBase (CNGBdb) under the accession number of N_000000002.1-N_ 000000085.1.
