# Supplementary material for: Mitochondrial genomes of the early land plant lineage liverworts (Marchantiophyta): conserved genome structure, and ongoing low frequency recombination
Source: BMC Genomics. 2019 Dec 9;20:953. doi: 10.1186/s12864-019-6365-y (PMC6902596; doi:10.1186/s12864-019-6365-y)
Supplement: Supplementary file 8 — Additional file 8: Figure S5. RNA editing site distributions on three gene alignments in a phylogenetic context. [file 12864_2019_6365_MOESM8_ESM.pdf]

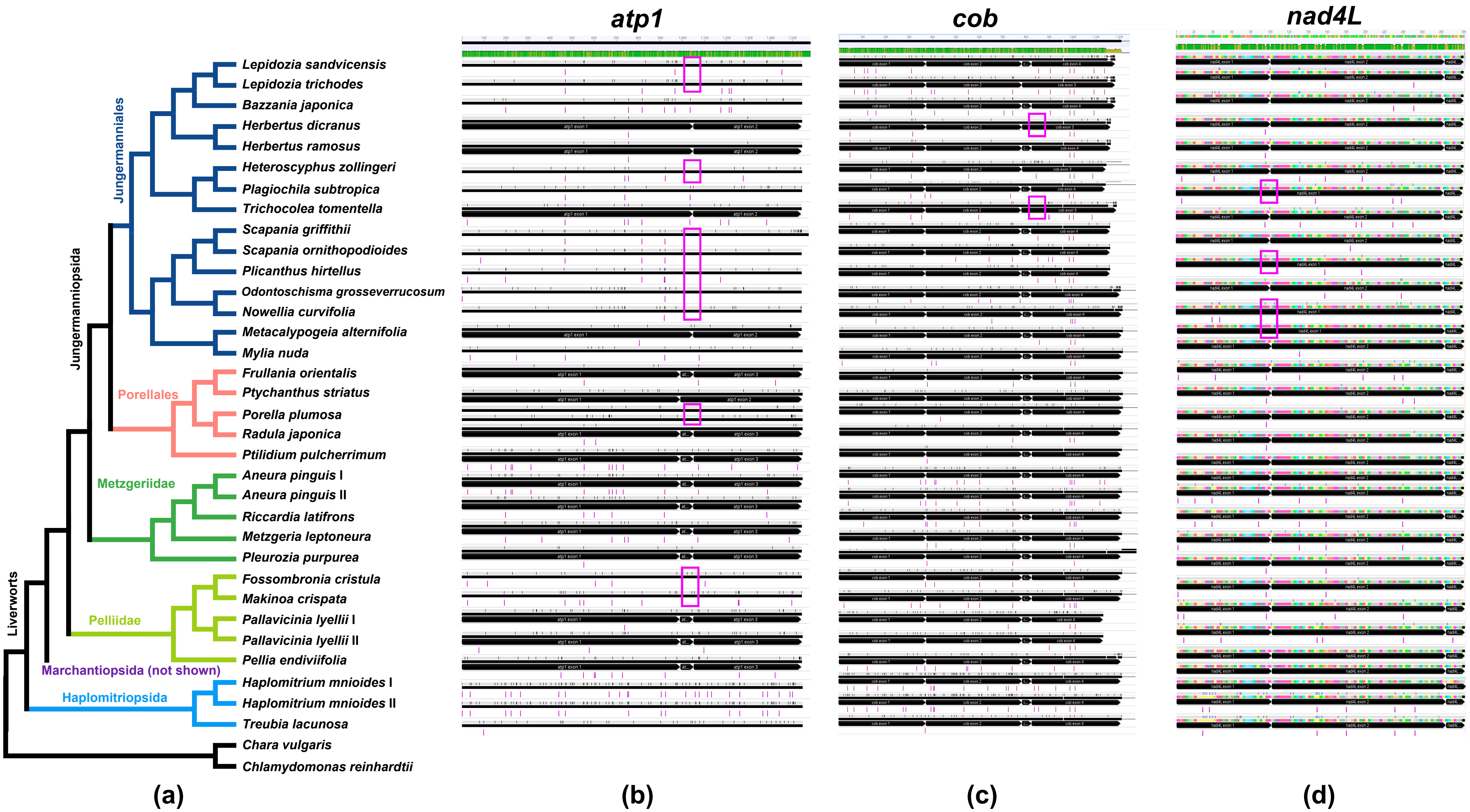

Figure S5. RNA editing site distributions on three gene alignments in a phylogenetic context. The short vertical lines in magenta represent the positions of the RNA editing sites, and the squares in magenta indicate possible retroprocessing events. (a) ML phylogenetic tree inferred from 128 organellar protein-coding genes of liverworts. for simplicity, the lineage of Marchantiopsida is not shown as accessions from this clade have no RNA editing sites detected; (b) the alignment of the coding region of *atp1* gene; (c) the alignment of the coding region of *cob* gene; (d) the alignment of the coding region of *nad4L* gene. These RNA editing data can be obtained from the draft mitochondrial genomes of liverworts deposited in the China National GeneBank DataBase (CNGBdb) under the accession number of N_000000002.1-N_000000085.1.
