# Supplementary material for: Mitochondrial genomes of the early land plant lineage liverworts (Marchantiophyta): conserved genome structure, and ongoing low frequency recombination
Source: BMC Genomics. 2019 Dec 9;20:953. doi: 10.1186/s12864-019-6365-y (PMC6902596; doi:10.1186/s12864-019-6365-y)
Supplement: Supplementary file 12 — Additional file 12: Figure S6. Phylogenetic trees of DSBR protein sequences from 125 Viridiplantae taxa inferred by Iqtree. [file 12864_2019_6365_MOESM12_ESM.pdf]

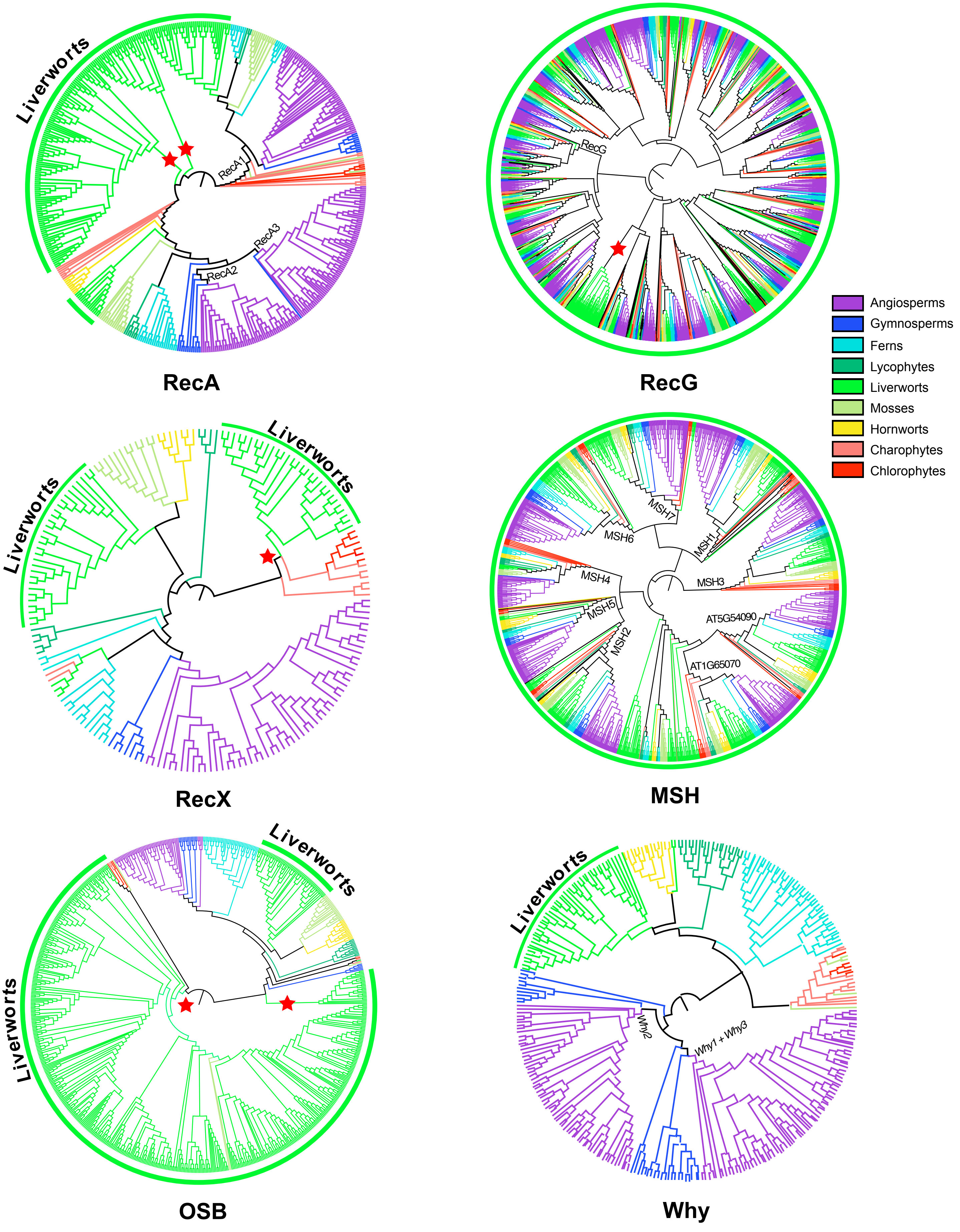

Figure S6. Phylogenetic trees of DSBR protein sequences from 125 Viridiplantae taxa inferred by IQtree. Taxa from the same phylogenetic group are colored the same. The red stars indicate liverwort specific DSBR protein subfamily expansions.
